# Update on the geographical distribution and prevalence of *Aedes aegypti* and *Aedes albopictus* (Diptera: Culicidae), two major arbovirus vectors in Cameroon

**DOI:** 10.1371/journal.pntd.0007137

**Published:** 2019-03-18

**Authors:** Armel N. Tedjou, Basile Kamgang, Aurélie P. Yougang, Flobert Njiokou, Charles S. Wondji

**Affiliations:** 1 Centre for Research in Infectious Diseases, Yaoundé, Cameroon; 2 Department of Animal Biology, Faculty of Sciences, University of Yaoundé I, Yaoundé, Cameroon; 3 Liverpool School of Tropical Medicine, Liverpool, United Kingdom; Centers for Disease Control and Prevention, Puerto Rico, UNITED STATES

## Abstract

**Introduction:**

Arboviral diseases including dengue are increasingly spreading in the tropical/subtropical world including Africa. Updated knowledge on the distribution and abundance of the major vectors *Aedes aegypti* and *Aedes albopictus* constitutes crucial surveillance action to prepare African countries such as Cameroon for potential arbovirus outbreaks. Here, we present a nationwide survey in Cameroon to assess the current geographical distribution and prevalence of both vectors including a genetic diversity profiling of *Ae*. *albopictus* (invasive species) using mitochondrial DNA.

**Methods:**

Immature stages of *Aedes* were collected between March and August 2017 in 29 localities across Cameroon following north-south and east-west transects. Larvae and pupae were collected from several containers in each location, reared to adult and morphologically identified. Genetic diversity of *Ae*. *albopictus* from 16 locations were analysed using Cytochrome Oxidase I gene (COI).

**Results:**

In total, 30,381 immature stages of *Aedes* with an average of 646.40±414.21 per location were identified across the country comprising 69.3% of *Ae*. *albopictus* and 30.7% of *Ae*. *aegypti*. Analysis revealed that *Ae*. *aegypti* is still distributed nation widely whereas *Ae*. *albopictus* is limited to the southern part, around 6°4’N. However, *Ae*. *albopictus* is the most prevalent species in all southern locations where both species are sympatric except in Douala where *Ae*. *aegypti* is predominant. This suggests that factors such as climate, vegetation, and building density impact the distribution of both species in Cameroon. Mitochondrial DNA analysis revealed a low genetic diversity in *Ae*. *albopictus* populations with a major common haplotype resulting in low haplotype diversity ranging from 0.13 to 0.65 and 0.35 for the total sample. Similarly, low nucleotide diversity was also reported varying from 0.0000 to 0.0017 with an overall index of 0.0008. This low genetic polymorphism is consistent with the recent introduction of *Ae*. *albopictus* in Cameroon.

**Conclusion:**

This updated distribution of arbovirus vectors across Cameroon will help in planning vector control programme against possible outbreak of arbovirus related diseases in the country.

## Author summary

*Aedes albopictus* and *Ae*. *aegypti* are the most important arbovirus vectors worldwide. *Ae*. *albopictus*, native of Asia, was recorded for the first time in early 2000s in Cameroon, central Africa. Previous studies performed a decade ago in Cameroon showed that *Ae*. *albopictus* has a geographical distribution limited to the south under 6°N. Whereas the native species *Ae*. *aegypti* was present across the country. To update our knowledge in this regards, a nationwide survey was performed in Cameroon to assess the current geographical distribution and prevalence of both vectors including a genetic diversity profiling of *Ae*. *albopictus* (invasive species) using mitochondrial DNA. Analysis revealed that *Ae*. *aegypti* is still distributed nation widely whereas *Ae*. *albopictus* is limited to the southern part, around 6°4’N. However, *Ae*. *albopictus* is the most prevalent species in all southern locations where both species are sympatric except in Douala where *Ae*. *aegypti* is predominant. This suggests that factors such as climate, vegetation and building density impact the distribution of both species in Cameroon. Mitochondrial DNA analysis revealed a low genetic diversity in *Ae*. *albopictus* populations with a major common haplotype detected in almost all locations. This study provides the relevant data that can be helpful to establish the vector surveillance of epidemic arbovirus vectors across the country.

## Introduction

*Aedes*-borne arboviral diseases such as dengue, Zika, and chikungunya are increasing global public health problems due to their rapid geographical spread and rising disease burden [1,2]. The viruses causing these infections are transmitted to vertebrates, including human, mainly by bites of infected mosquito belonging to the *Aedes* genus. However, transmission by blood transfusion and/or by sexual contacts have also been reported in Zika virus [3]. Two main epidemic vectors of these arboviral diseases, *Aedes aegypti* Linnaeus 1762 and *Aedes albopictus* (Skuse) 1894, have different origins. *Aedes aegypti*, originating from African forests, is currently found in most tropical and subtropical regions worldwide [4,5]. While *Ae*. *albopictus* originating from South Asia forest has invaded all the five continents during the past 30–40 years [6,7]. This rapid spread of *Ae*. *albopictus* has been mainly facilitated by international commercial exchanges notably international trade of used tires as previously demonstrated [8], and strong ecological and physiological plasticity allowing it to thrive in a wide range of climates and habitats [9]. This invading species was recorded for the first time in central Africa in Cameroon in the early 2000s [10] and became the dominant species over the native species *Ae*. *aegypti* in sympatric areas [11]. Interestingly, dengue, Zika, and chikungunya, which were considered to be scarce in central Africa, are emerging in several urban foci simultaneously with the introduction of *Ae*. *albopictus* [12–14] suggesting a modification of epidemiology of arboviral diseases. During the last two decades, the circulation of arboviral diseases has been well documented in Cameroon [14–20], but nationwide studies have not been undertaken to get an accurate picture of distribution of these viruses across the country. Meanwhile, the seroprevalence of dengue in general population was assessed in three main cities of Cameroon located in different ecological settings in 2006/2007. This study revealed that 61.4% of people tested had immunoglobulin (Ig) G and 0.3% IgM in Douala, 24.2% IgG and 0.1% IgM in Garoua and 9.8% IgG and no IgM in Yaoundé [21]. More recently, another study in blood donors in population from six cities across Cameroon revealed that the overall seroprevalence of Zika was low around 5%, peaking at 10% and 7.7% in Douala and Bertoua respectively and only 2% in Ngaoundere and Maroua [22]. Indeed, *Ae*. *albopictus* has been detected as the main vector in Gabon during concurrent dengue/chikungunya outbreak in 2007 [23,24]. Both *Ae*. *aegypti* and *Ae*. *albopictus* have been found infected by chikungunya virus during a large outbreak that occurred in the Republic of the Congo in 2011 [25].

The coexistence of both *Ae*. *aegypti* and *Ae*. *albopictus* has been well documented in several regions throughout the world. In central Africa, both species often share the same larval habitats, although *Ae*. *albopictus* preferred man-made containers such as used tires and discarded tanks surrounded by the presence of vegetation whereas *Ae*. *aegypti* preferred larval habitats located in a neighborhood with a high building density [26,27]. Competitive displacement between both species has also been well studied in South America and South East Asia revealing that the invasive species often have the competitive advantage over the native species. For example, in Asia, the overall advantage of *Ae*. *aegypti* over the resident species *Ae*. *albopictus* has been reported [28,29]. In contrast, replacement of *Ae*. *aegypti* by invasive species *Ae*. *albopictus* was reported in south-eastern USA and Brazil [30–32] and was suspected in Réunion [33] and Mayotte [34]. In central Africa, the dominance of the invading species *Ae*. *albopictus* over the native species *Ae*. *aegypti* was also reported in the locations where both species were found together [24,35]. The last study conducted in Cameroon on the geographical distribution of *Ae*. *aegypti* and *Ae*. *albopictus* date more than 10 years. The results of the study revealed that *Ae*. *aegypti* was present across the country while the distribution of *Ae*. *albopictus* was limited to the south, under 6°N [36,37]. Climatic limitations and the dynamics of invasion have been used to explain the absence of *Ae*. *albopictus* beyond 6°N but no temporal study has been performed to assess the dynamic of this distribution and establish whether this species could spread further northwards increasing the risk of arbovirus transmission. *Ae*. *albopictus* being more competent to transmit dengue, chikungunya and probably Zika in central Africa [11], it is important to properly define the vector composition in this region to adequately prepare for future outbreaks. This requires updating the data on the geographical distribution and prevalence of *Ae*. *aegypti* and *Ae*. *albopictus* country-wide. Furthermore, analysis of the genetic diversity of the invasive species is also needed to characterize the population demographic history of this species in Cameroon since its introduction. Previous studies analysing the genetic diversity of *Ae*. *albopictus* in Central Africa using the cytochrome oxidase subunit I (COI) gene had revealed a low polymorphism and showed that Cameroonians’ population are related to tropical rather than temperate or subtropical out-groups [9,35]. It remains to establish how this genetic diversity has evolved in the past decade and whether the population of this species has experienced demographic events such as expansion, new colonisation or bottleneck. Here, we present an extensive and nationwide analysis of the current geographical distribution and prevalence of *Ae*. *aegypti* and *Ae*. *albopictus* in Cameroon as well as the genetic diversity of the invading *Ae*. *albopictus* species to improve the entomological surveillance of these vectors.

## Materials and methods

### Ethics statement

This study was approved by the Cameroonian national ethics committee for human health research N°2017/05/911/CE/CNERSH/SP. Oral consent to inspect the potential breeding sites was obtained in the field in household or garage owners.

### Study sites

**Fig 1:**
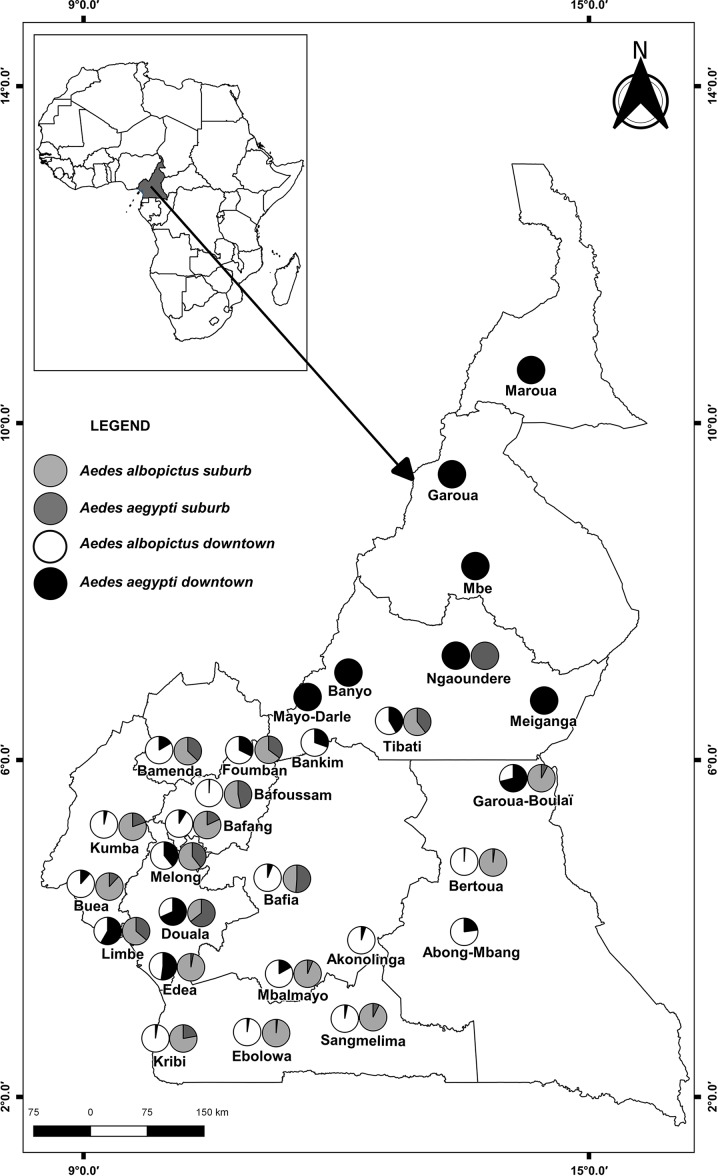
Geographic distribution of *Ae*. *aegypti* and *Ae*. *albopictus* in Cameroon, March-August 2017.

Mosquitoes were collected in 28 locations across Cameroon, a central African country located between 1°40–13°05N and 8°30–16°10E (Table 1 and Fig 1), following north-south and east-west transects. Cameroon is characterized by a broad range of biotopes varying from the sudano-sahelian climate in the far north to the equatorial guinean forest climate in the south with strong local climate heterogeneities due to huge variations in altitude (0 to 4,000 m above sea level on Mount Cameroon) [36]. The annual precipitation rate vary from 400 mm in the arid areas to 10,000 mm at the foot of mount-Cameroon (>4,000m above sea level). Annual temperatures and relative humidity vary between 18°C to 28°C and 85% to 45%, respectively [38] (Table 1). Mosquito sampling focused mainly on urban settlements that spread along the trade routes throughout the country. This is because invading *Aedes* species has been directly linked to human activities [39] and important outbreaks usually occur in urban settings.

**Table 1:** Environmental characteristics of different prospected sites in Cameroon.

Localities	Lat (N)	Long (E)	Climate	Vegetation	Population
**Maroua**	10°59'	14°32'	Sahelian Sudano climate	Dense herbaceous savanna	33,0410
**Garoua**	9°32	13°39'	Sudanian tropical basin	Tree savanna with shrub deciduous	26,5583
**Mbe**	7°86'	13°59'	Tropical humid altitude	Tree savanna with shrub deciduous	17,478
**Ngaoundere**	7°33’	13°56'	Tropical humid altitude	Wooded savannah deciduous	26,2747
**Banyo**	6°74'	11°80'	Tropical humid altitude	Wooded savannah deciduous	93,880
**Meiganga**	6°51'	14°29'	Tropical humid altitude	Post-forest transition and savannah	88,745
**Tibati**	6°47'	12°61'	Tropical altitude	Post-forest transition and savannah	72,081
**Mayo-Darle**	6°46'	11°54'	Tropical humid altitude	Wooded savannah deciduous	23,054
**Bankim**	6°08'	11°48'	Tropical altitude	Savannah forest mosaic	70,132
**Bamenda**	5°96'	10'15'	Equatorial mountain monsoon climate	Savannah forest mosaic	322,889
**Garoua-Boulai**	5°89'	14°54'	Guinean subequatorial climate	Savannah forest mosaic	41,388
**Foumban**	5°72'	10°90'	Equatorial mountain monsoon climate	Savannah forest mosaic	130,292
**Bafoussam**	5°48'	10°42'	Equatorial mountain monsoon climate	Savannah forest mosaic	98,339
**Bafang**	5°16'	10°18'	Equatorial mountain monsoon climate	Wooded savannah deciduous	34,941
**Melong**	5°12'	9°95'	Equatorial monsoon with higher pluviometry	Wet dense forest	54,279
**Bafia**	4°75'	11°22'	Subequatorial climate under shelter	Wet dense forest	72,717
**Mbalmayo**	3°30'	11°30'	Guinean subequatorial climate	Wet dense forest	62,808
**Kumba**	4°63'	9°44'	North Coast Equatorial	Wet dense forest	166,331
**Bertoua**	4°57'	13°67'	Guinean subequatorial climate	Savannah forest mosaic	111,986
**Buea**	4°15'	9°26'	North Coast Equatorial	Mountain forest	131,325
**Douala**	4°05'	9°76'	Equatorial monsoon with higher pluviometry	mangrove swamp and wooded	1,926,513
**Limbe**	4°02'	9°19'	Equatorial monsoon with higher pluviometry	submontane forest	118,210
**Abong-Mbang**	3°97'	13°17'	Guinean subequatorial climate	Wet dense forest	29,005
**Edea**	3°79'	10°13'	Equatorial monsoon with higher pluviometry	Wet dense forest	8,8481
**Akonolinga**	3°78'	12°24'	Guinean subequatorial climate	Wet dense forest	47,561
**Kribi**	2°94'	9°91'	Guinean subequatorial climate	Wet dense forest	93,246
**Sangmelima**	2°93'	11°59'	Guinean subequatorial climate	Wet dense forest	91,740
**Ebolowa**	2°92'	11°15'	Guinean subequatorial climate	Wet dense forest	118,267

### Mosquito collection and identification

Immature stages of mosquitoes were collected from mid-March to August 2017 corresponding to the rainy season. In most locations located under 6°N where both *Ae*. *aegypti* and *Ae*. *albopictus* were previously detected [36,37], collections in each town were performed in both the downtown and suburb to assess habitat segregation of both species according to building density and vegetation as demonstrated previously [27,35]. For collection located above 6°N, investigations were performed across the city and pooled together. In each selected location, all the containers with water (potential breeding sites) were inspected and container with at least one larva and/or pupa suspected to belong to *Aedes* genus (positive breeding sites) was recorded. The number of potential and positive containers were reported. The immature stages were collected, transported to the insectary and pooled together according to the environment (downtown vs. suburban) and location. They were maintained in the insectary until adult emergence. The emerged adults were identified under a binocular magnifying glass by the morphological criteria previously described [40–42], numbered, pooled in a breeding cage according to species and location, and further reared in the controlled conditions (27°C +/- 2°C; relative humidity 80% +/-10%) until generation 1 (G1) for further analysis. G_0_ adults were stored at -20°C for further molecular and genetic analyses. The prevalence of *Ae*. *aegypti* and *Ae*. *albopictus* was compared per environment and per location and statistically analysed using the Chi-square test. The Cameroon data Shape files were downloaded from the Global Administrative Areas (GADM) version 2.8 web site and the global positioning system coordinates were projected according to the WGS 84-EPSG 4326 system and the proportion of each *Aedes* species was generated with the QGIS version 3.4.1-Madeira software.

### Mitochondrial DNA sequencing and analysis

The total DNA was extracted from 20 G_0_ individuals of *Ae*. *albopictus* collected in 17 sites (including samples from Yaoundé) using the Livak method as previously described **[43]**.

DNA extracts from each locality were used as templates to amplify a 700-bp fragment of MtCOI gene. The sequences of the primers used are albCOIF 5’-TTTCAACAAATCATAAAGATATTGG-3’ and albCOIR 5’- TAAACTTCTGGA TGACCAAAAAATCA-3’ [44]. Polymerase chain reaction (PCR) amplification was performed using a Gene Touch thermal cycler (Bulldog Bio, Portsmouth, USA) as described previously [44]. Amplicons from the PCR were analysed by agarose gel electrophoresis stained with Midori green and visualized under UV light. Fifteen PCR products from each locality were purified using Exo-SAP protocol according to manufacturer recommendations and sequenced directly.

Sequences were visualized and corrected manually when necessary using BioEdit software version 7.0.5.3 and aligned using Clustal W [45]. Sequences were numbered based on the reference sequence downloaded from GenBank (Accession number KU738429.1) that originated from China. The number of haplotypes (h), the number of polymorphism sites (S), haplotype diversity (Hd), nucleotide diversity (π), were computed with DnaSP 5.10 [46]. The statistical tests of Tajima [47], Fu and Li [48] were estimated with DnaSP in order to establish non-neutral evolution and deviation from mutation-drift equilibrium. Different haplotypes detected were compared to previous COI region sequences published in GenBank from populations that originated from China, USA, Singapore, Thailand, Italy, Japan and Congo [49,50]. These COI sequences were used to construct the maximum likelihood phylogenetic tree using MEGA 7.0 [51]. Genealogical relationship between haplotype detected across Cameroon was assessed using TCS [52] and tcsBU [53] software.

## Results

### Larval habitats prospected per location

A total of 4,054 potential breeding sites was inspected in 28 locations across Cameroon, out of which 1,103 (27.20%) were found containing immature stages of *Aedes* (positive breeding sites). Detected breeding sites were grouped in six categories: used tires, discarded tanks; miscellaneous; water storage tanks; natural and recycled tires commonly used by the locals to protect wells (Table 2). Used tires were the most potential breeding sites discovered at 87.53% (3,545/4,050) and mostly infested (85.31%, 941/1,103) by *Aedes* larvae. The prevalence of other breeding sites was very low with 0.45% (5/1,103) in natural breeding sites, 3.0% (33/1,103) in tires covering water wells, 1.45% (16/1,103) in miscellaneous and 5.07% (56/1,103) in discarded tanks (Table 2).

**Table 2:** Containers prospected per site in Cameroon, mid-March to August 2017.

Location	Used tires n (%)	Tires on well n (%)	Water storage n (%)	Discarded tanks n (%)	Natural n (%)	Miscellaneous n (%)	Total n (%)
Maroua	72 (43.1)	0 (NC)	2 (50.0)	1 (100)	0 (NC)	2 (50)	77 (44.2)
Garoua	136 (27.2)	0 (NC)	2 (50)	2 (100)	0 (NC)	3 (33.3)	143 (29.4)
Mbe	22 (22.7)	0 (NC)	5 (20)	7 (100)	2 (50)	0 (NC)	36 (38.9)
Meiganga	54 (27.8)	0 (NC)	38 (5.3)	0 (NC)	0 (NC)	9 (44.4)	99 (21.2)
Banyo	68 (14.7)	0 (NC)	0 (NC)	4 (25)	0 (NC)	7 (100)	79 (22.8)
Mayo-Darle	119 (13.4)	0 (NC)	0 (NC)	0 (NC)	0 (NC)	1 (100)	120 (14.2)
Bankim	150 (24.7)	8 (87.5)	0 (NC)	4 (50)	0 (NC)	0 (NC)	162 (28.4)
Ngaoundere downtown	73 (42.8)	0 (NC)	0 (NC)	19 (10.5)	0 (NC)	0 (NC)	92 (35.9)
Ngaoundere suburban	43 (27.9)	0 (NC)	0 (NC)	3 (66.7)	0 (NC)	7 (100)	53 (39.6)
Tibati downtown	153 (28.8)	0 (NC)	0 (NC)	4 (50)	0 (NC)	7 (42.9)	164 (29.9)
Tibati suburban	71 (35.2)	12 (58.3)	0 (NC)	23 39.1)	0 (NC)	2 (0)	108 (37.9)
Foumban downtown	113 (20.4)	0 (NC)	0 (NC)	9 (66.7)	0 (NC)	6 (66.7)	128 (25.8)
Foumban suburban	85 (23.5)	0 (NC)	0 (NC)	1 (0)	0 (NC)	0 (NC)	86 (23.3)
Bafoussam downtown	358 (2.2)	0 (NC)	2 (0)	2 (0)	0 (NC)	3 (0)	365 (2.2)
Bafoussam suburban	237 (10.6)	0 (NC)	0 (NC)	0 (NC)	0 (NC)	3 (0)	240 (10.4)
Bafang downtown	33 (54.5)	4 (100)	0 (NC)	3 (0)	0 (NC)	1 (0)	41 (53.6)
Bafang suburban	6 (66.7)	0 (NC)	8 (12.5)	9 (11.1)	0 (NC)	20 (25)	43 (25.6)
Bamenda downtown	241 (9.1)	0 (NC)	0 (NC)	1 (0)	0 (NC)	2 (0)	244 (9.0)
Bamenda suburban	98 (15.3)	0 (NC)	0 (NC)	0 (NC)	0 (NC)	0 (NC)	98 (15.3)
Bafia downtown	82 (26.8)	0 (NC)	0 (NC)	2 (0)	0 (NC)	1 (0)	85 (25.9)
Bafia suburban	68 (8.8)	0 (NC)	4 (0)	30 (13.3)	1 (100)	27 (0)	130 (8.4)
Mbalmayo downtown	36 (22.2)	0 (NC)	0 (NC)	0 (NC)	0 (NC)	1 (100)	36 (25.0)
Mbalmayo suburban	65 (58.5)	0 (NC)	0 (NC)	0 (NC)	0 (NC)	0 (NC)	65 (58.5)
Akonolinga suburban	31 (22.6)	0 (NC)	0 (NC)	3 (100)	0 (NC)	12 (66.7)	46 (39.1)
Ebolowa downtown	57 (52.6)	0 (NC)	1 (100)	2 (50)	0 (NC)	2 (50)	62 (53.2)
Ebolowa suburban	37 (59.5)	0 (NC)	0 (NC)	4 (25)	2 (0)	12 (41.7)	55 (50.9)
Sangmelima downtown	64 (46.9)	0 (NC)	1 (100)	3 (100)	0 (NC)	4 (75)	72 (51.4)
Sangmelima suburban	15 (33.3)	0 (NC)	5 (60)	6 (50)	0 (NC)	12 (50)	36 (52.8)
Garoua-Boulai downtown	117 (25.6)	2 (50)	1 (100)	1 (0)	0 (NC)	2 (0)	123 (26.01)
Garoua-Boulai suburban	2 (100)	17 (47.05)	0 (NC)	12 (8.3)	0 (NC)	0 (NC)	31 (35.5)
Bertoua downtown	62 (61.3)	2 (50)	0 (NC)	2 (50)	0 (NC)	0 (NC)	66 (60.6)
Bertoua suburban	56 (42.9)	6 (33.3)	0 (NC)	1 (0)	0 (NC)	0 (NC)	63 (41.)3
Abong-Mbang	31 (58.1)	1 (100)	0 (NC)	3 (0)	3 (100)	1 (100)	39 (59.0)
Kumba downtown	35 (25.7)	0 (NC)	0 (NC)	7 (57.1)	0 (NC)	3 (33.3)	45 (33.3)
Kumba suburban	20 (55.0)	1 (100)	3 (33.3)	4 (25.0)	1 (0)	7 (42.9)	36 (47.2)
Limbe downtown	40 (65.0)	0 (NC)	0 (NC)	0 (NC)	0 (NC)	0 (NC)	40 (65.0)
Limbe suburban	95 (47.4)	0 (NC)	0 (NC)	0 (NC)	0 (NC)	2 (100)	97 (48.5)
Buea downtown	88 (23.9)	0 (NC)	0 (NC)	2 (0)	0 (NC)	3 (0)	93 (22.6)
Buea suburban	100 (26.0)	0 (NC)	0 (NC)	0 (NC)	0 (NC)	0 (NC)	100 (26.0)
Melong downtown	70 (34.3)	2 (50)	2 (0)	5 (20)	0 (NC)	11 (63.6)	90 (36.7)
Melong suburban	52 (32.7)	0 (NC)	0 (NC)	2 (0)	0 (NC)	21 (14.3)	75 (26.7)
Douala downtown	36 (91.7)	0 (NC)	0 (NC)	0 (NC)	0 (NC)	0 (NC)	36 (91.7)
Douala suburban	100 (26)	0 (NC)	0 (NC)	1 (100)	0 (NC)	0 (NC)	101 (26.73)
Edea downtown	24 (87.5)	0 (NC)	0 (NC)	0 (NC)	0 (NC)	0 (NC)	24 (87.5)
Edea suburban	30 (36.66)	0 (NC)	0 (NC)	0 (NC)	0 (NC)	0 (NC)	30 (30.66)
**Total**	**3,545 (26.5)**	**40 (82.5)**	**63 (19.04)**	**176 (31.8)**	**9 (55.6)**	**188 (39.4)**	**4,054 (27.2)**

N, number of containers found with water; (%), the percentage of positive containers; NC, not computed.

### Distribution and prevalence of *Ae*. *aegypti* and *Ae*. *albopictus* across the country

30,381 immature stages of *Ae*. *albopictus* and *Ae*. *aegypti* species were collected in 29 localities between mid-March and August 2017 in Cameroon (Table 3). Several other species were found in association with *Ae*. *aegypti* and *Ae*. *albopictus*. These include *Aedes simpsoni* Theobald 1905, *Aedes vittatus* Bigot 1861, *Anopheles gambiae* s.l. Giles 1902, *Culex tigripes* De Grandpré and De Charmoy 1900, *Culex pipiens quinquefasciatus* Say, 1823, *Culex perfuscus* Edwards 1914, *Culex duttoni* Theobald 1901, *Culex antennatus* Beker 1903, *Culex* sp Linnaeus 1758, *Eretmapodites brevipalpis* Ingram and De Meillon 1927, and *Toxorhynchites brevipalpis* Ribeiro 1991.

**Table 3:** Relative abundance of *Ae*. *albopictus* and *Ae*. *aegypti* across the prospected location in Cameroon.

Location	*Ae*. *albopictus* n (%)	*Ae*. *aegypti* n (%)
Kumba downtown	717 (96.24)	28 (3.76)
Kumba suburban	157 (80.51)	38 (19.49)
Buea downtown	743 (88.45)	97 (11.55)
Buea suburban	697 (88.23)	93 (11.77)
Limbe downtown	313 (41.62)	439 (58.38)
Limbe suburban	863 (63.74)	491 (36.26)
Melong downtown	347 (60.66)	225 (39.34)
Melong suburban	156 (60.47)	102 (39.53)
Douala downtown	319 (31.71)	687 (68.29)
Douala suburban	214 (35.73)	385 (64.27)
Edea downtown	164 (47.81)	179 (52.19)
Edea suburban	168 (97.11)	5 (2.89)
Bamenda downtown	245 (83.62)	48 (16.38)
Bamenda suburban	247 (62.85)	146 (37.15)
Bafoussam downtown	272 (99.27)	2 (0.73)
Bafoussam suburban	294 (53.55)	255 (46.45)
Bafang downtown	1032 (91.17)	100 (8.83)
Bafang suburban	182 (81.98)	40 (18.02)
Foumban downtown	442 (67.69)	211 (32.31)
Foumban suburban	335 (64.67)	183 (35.33)
Bafia downtown	844 (93.99)	54 (6.01)
Bafia suburban	306 (48.73)	322 (51.27)
Mbalmayo downtown	504 (83.17)	102 (16.83)
Mbalmayo suburban	1055 (93.86)	69 (6.14)
Akonolinga*	1449 (94.89)	78 (5.11)
Ebolowa downtown	305 (97.44)	8 (2.56)
Ebolowa suburban	566 (98.43)	9 (1.57)
Sangmelima downtown	679 (96.86)	22 (3.14)
Sangmelima suburban	437 (92.78)	34 (7.22)
Kribi downtown	495 (97.44)	13 (2.6)
Kribi suburban	1597 (77.94)	452 (22.06)
Abong-Mbang*	656 (77.18)	194 (22.82)
Bertoua downtown	769 (99.1)	7 (0.9)
Bertoua suburban	867 (97.97)	18 (2.03)
Garoua-Boulaï downtown	59 (28.92)	145 (71.08)
Garoua-Boulai suburban	430 (92.47)	35 (7.53)
Tibati downtown	733 (58.5)	520 (41.5)
Tibati suburban	359 (60.44)	235 (39.56)
Bankim*	1031 (69.57)	451 (30.43)
Ngaoundere downtown	0	556 (100)
Ngaoundere suburban	0	147 (100)
Mbe*	0	249 (100)
Garoua*	0	812 (100)
Maroua*	0	146 (100)
Meiganga*	0	484 (100)
Banyo*	0	292 (100)
Mayo-Darle*	0	125 (100)
**Total**	**21,048 (69.28)**	**9,333 (30.72)**

n, number of adult mosquitoes identified; %, percentage

*, mosquitoes collected across the city and pool together.

*Aedes aegypti* was found across the country in all the sites investigated whereas *Ae*. *albopictus* distribution was limited to the southern part of the country under 6°4’N (Fig 1 and Table 3). Overall, *Ae albopictus* was more prevalent (69.28%) than *Ae*. *aegypti* (30.72%)(Table 3). In all the locations in which both species were found together *Ae*. *albopictus* was found to be more abundant except in Douala where *Ae*. *aegypti* was predominant in downtown and suburban. When analyses were done according to the environment (suburban vs downtown) in each location, *Ae*. *albopictus* was found to be the dominant species in the suburban and downtown in all the sympatric areas except in Garoua-Boulai, Douala, Limbe, and Edea where *Ae*. *aegypti* was predominant in downtown (Table 3). Analysis also revealed that in some locations such as Bertoua, Kribi, Sangmelima, Ebolowa and Bafoussam, *Ae*. *albopictus* is highly prevalent in all areas and nearly excluding the native species which sometimes represents less than 3%.

### Genetic diversity of *Ae*. *albopictus* with Mitochondrial DNA

**Fig 2:**
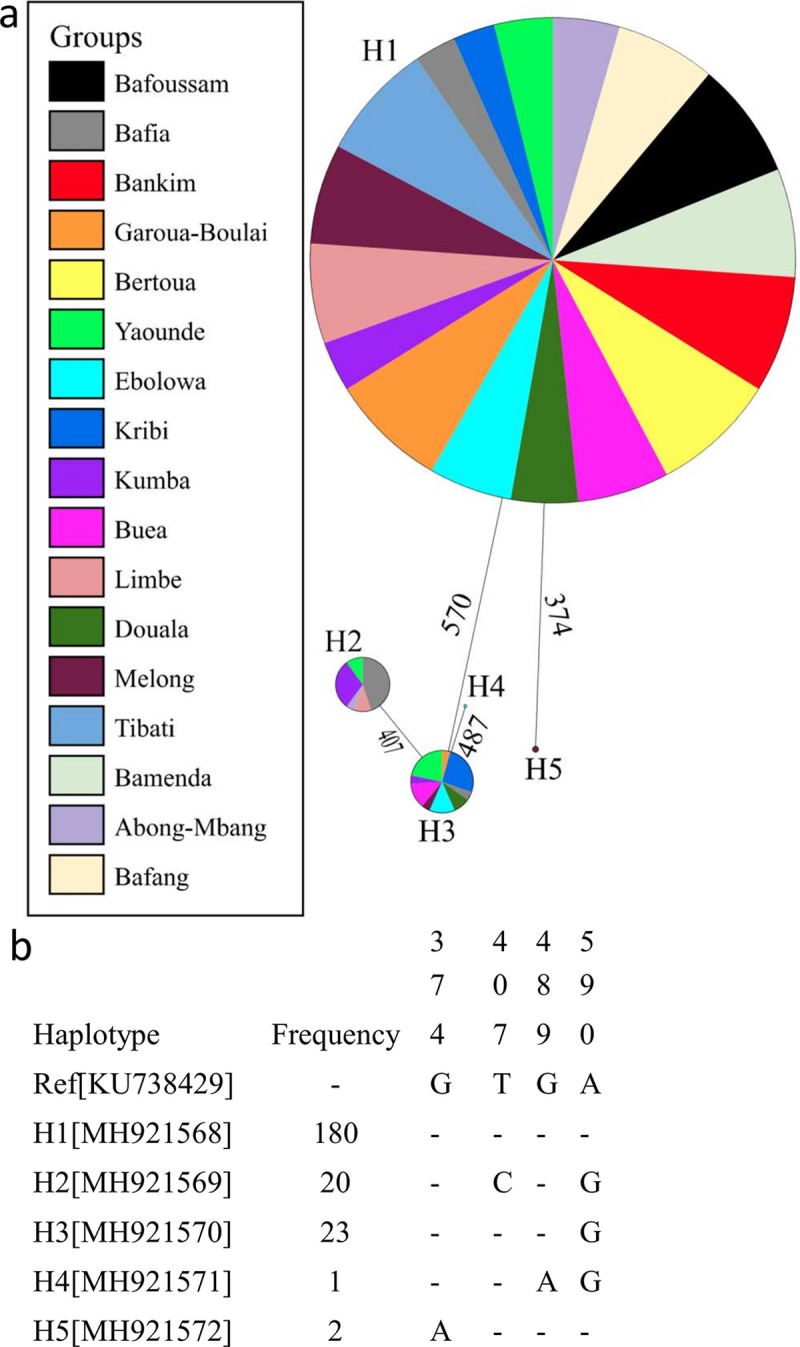
Genetic diversity of the COI gene across Cameroonian populations of *Ae*. *albopictus*. a, Haplotype network showing the genealogic relationship between five haplotypes detected across Cameroon. b, COI haplotypes found across Cameroon. Only polymorphic positions are shown and are numbered with reference (Ref) to the published *Ae*. *albopictus* sequences for COI (JF309317; China). Dots represent identity with respect to the reference.

**Fig 3:**
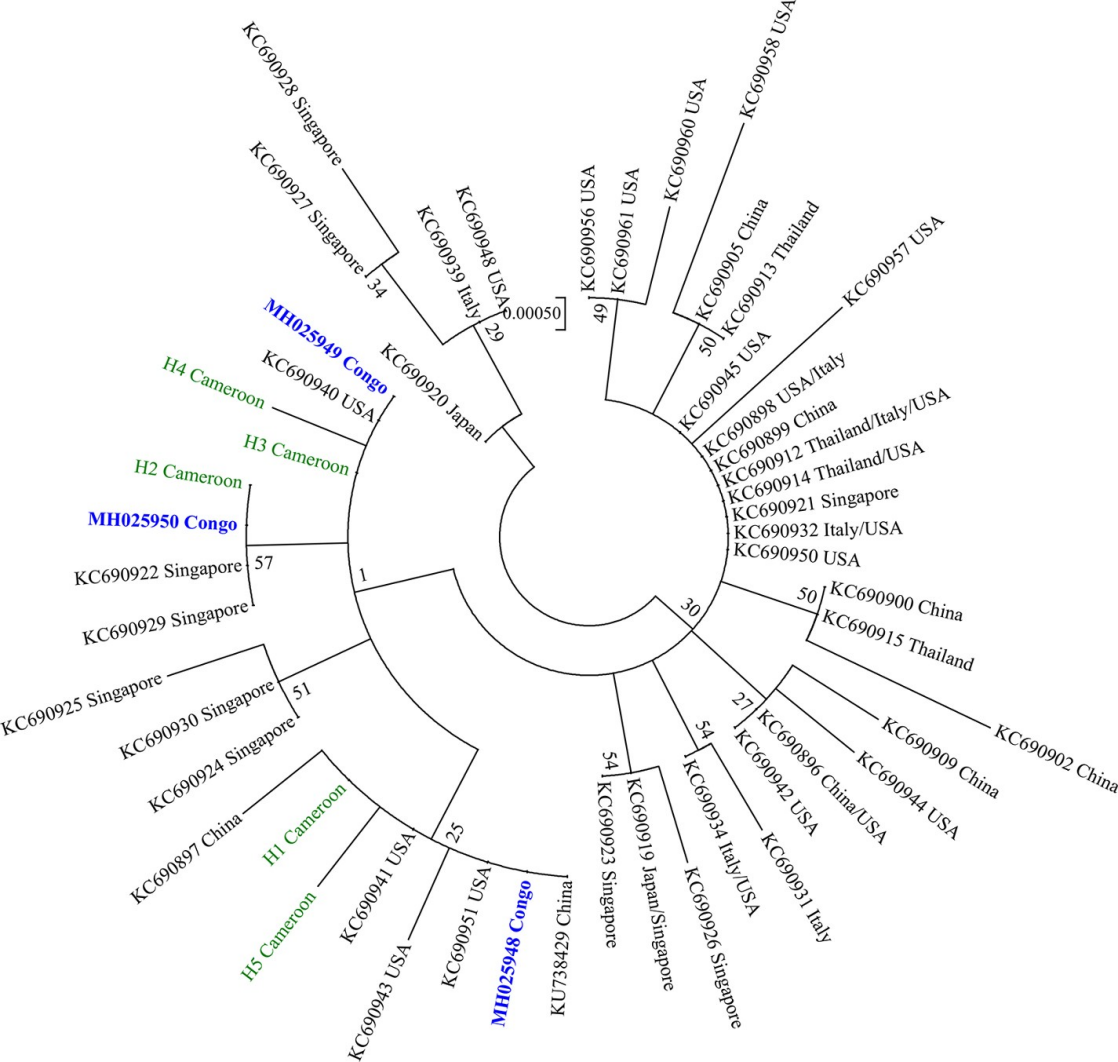
Molecular phylogenetic analysis of *Ae*. *albopictus* by maximum likelihood method. The evolutionary history was inferred by using the Maximum Likelihood method based on the Tamura 3-parameter model. The tree is drawn to scale, with branch lengths measured in the number of substitutions per site. There was a total of 636 positions in the final dataset.

In total, 226 individuals of *Ae*. *albopictus* from 17 localities throughout Cameroon were analysed with the mtCOI gene (Table 4). Sequences analysed based on 636 nucleotides revealed a low polymorphism, with four substitution sites defining five haplotypes resulting in low haplotype diversity (hd) ranging from 0.13 to 0.65 with overall hd of 0.32. Similarly, low nucleotide diversity (π) index was recorded varying from 0.0000 to 0.0017 with 0.00075 for total sample. The predominant haplotype H1 (79.7% in total sample) was recorded in all the locations with frequency ranging from 35.7% to 100% (Fig 2, S1 Table). This haplotype was also the most prevalent in almost all locations excepted in Bafia and Kribi where it is rather H2 (57.1%) and H3 (54.5%) haplotypes, respectively (S1 Table). The predominant H1 haplotype matches perfectly with the reference sequence downloaded from GenBank originating from China (KU738429.1) and corresponds to the predominant haplotype detected recently in a neighbouring country, the Republic of the Congo [50]. All the three haplotypes isolated previously in the Republic of the Congo were also detected with the same primer sets in Cameroon. Analysis of haplotype network revealed that each haplotype is isolated from the others by one mutational step. Overall, all the Tajima statistics estimated were negatives (D = -0.43, D* = -0.43, Fs = -0.93, and F* = -040), but not statistically significant (Table 4). Phylogenetic analysis of the 636bp fragment showed that Cameroonian and Congolese *Ae*. *albopictus* populations have the same origin as they cluster together on the Maximum likelihood tree (Fig 3). Nucleotide sequences of five haplotypes detected across Cameroon have been deposited in the GenBank database (accession numbers: MH921568, MH921569, MH921570, MH921571 and MH921572).

**Table 4:** Genetic diversity indices in *Aedes albopictus* from Cameroon.

Location	N	Hp	S	HpD	π (k)	D	D*	Fs	F*
Abong-Mbang	9	H1, H2	2	0.22	0.0007 (0.444)	-1.36	-1.51	0.67	-1.50
Bafang	13	H1, H2	2	0.15	0.0005 (0.308)	-1.47	-1.78	0.36	-1.75
Bafoussam	14	H1	0	NC	0.0000 (0.000)	NC	0.00	0.00	NC
Bamenda	13	H1	0	NC	0.0000 (0.000)	NC	0.00	0.00	NC
Bankim	14	H1	0	NC	0.0000 (0.000)	NC	0.00	0.00	NC
Bertoua	15	H1	0	NC	0.0000 (0.000)	NC	0.00	0.00	NC
Bafia	14	H1, H2, H3	2	0.58	0.0016 (1.022)	1.70	0.94	0.91	1.17
Buea	14	H1, H3	1	0.36	0.0006 (0.363)	0.32	0.72	0.64	0.64
Douala	10	H1, H3	1	0.36	0.0006 (0.355)	0.015	0.80	0.42	0.63
Ebolowa	14	H1, H3, H4	2	0.47	0.0009 (0.582)	-0.20	-0.45	-0.21	-0.40
Garoua-Boulai	15	H1, H3	1	0.13	0.0002 (0.133)	-1.16	-1.43	-0.65	-1.41
Kribi	11	H1, H3	1	0.55	0.0009 (0.545)	1.44	0.78	1.14	0.96
Kumba	13	H1, H2, H3	2	0.62	0.0017 (1.077)	1.88	0.95	0.93	1.23
Limbe	14	H1	2	0.26	0.0008 (0.527)	-0.44	0.94	1.25	0.60
Melong	15	H1, H3, H5	2	0.36	0.0006 (0.381)	-1.00	-0.48	-0.92	-0.64
Tibati	14	H1	0	NC	0.0000 (0.000)	NC	0.00	0.00	NC
Yaounde	14	H1, H2, H3	2	0.65	0.0013 (0.802)	0.75	0.93	0.40	0.92
**Total**	**226**	**5**	**4**	**0.35**	**0.0008 (0.503) (0.503)**	**-0.43**	**-0.43**	**-0.93**	**-0.51**

N: Number of sequences analysed; S: Number of segregating sites; π: Nucleotide diversity per site; k: Average number of nucleotide differences; Hp: Number of haplotypes; HpD: Haplotype diversity; Fs: Fu’s Statistic; D: Tajima’s statistic; D* and F*: Fu and Li’s statistics NC: Not Computed.

## Discussion

This study presents an extensive profiling of the prevalence and geographical distribution of *Ae*. *albopictus* and *Ae*. *aegypti* in Cameroon updating data generated more than 10 years ago. This current report reveals that *Ae*. *albopictus* distribution continues to be restricted to the southern part of the country, around 6°N latitude while *Ae*. *aegypti* is found throughout the country. The predominance of the invading species (*Ae*. *albopictus*) over the native species (*Ae*. *aegypti)* is also reported in almost all locations where both species are sympatric.

The current distribution of *Ae*. *aegypti* and *Ae*. *albopictus* is similar to the previous distribution reported in Cameroon in 2003 [36] and 2007 [37] and in the Central African Republic in 2012 [35] where the distribution of *Ae*. *albopictus* was shown to be restricted to the South around 6°N. This boundary has been suggested to be due to the unfavourable climatic conditions for the establishment of this species in the northern part of central Africa above 6°5’N rather than the dynamics of invasion process that is still ongoing as suggested previously [36]. Indeed, mean annual temperatures in Cameroon vary between 20 to 28° C and increase from the south towards the north. Although, both *Ae*. *aegypti* and *Ae*. *albopictus* have desiccant-resistant eggs, previous studies showed that *Ae*. *aegypti* eggs are more tolerant to high temperatures than those of *Ae*. *albopictus* [54].

Consequently, the climatic conditions in the southern part of Cameroon (i.e., equatorial climate, average annual temperature <26.5°C) are favourable to the development of *Ae*. *albopictus*. A higher prevalence of *Ae*. *albopictus* was observed in some sympatric areas in both suburban and downtown environment, which is in contrast to the previous results in central Africa. *Ae*. *aegypti* has often been the prevalent species in downtown with high building density whereas *Ae*. *albopictus* was predominant in suburban area surrounded by vegetation [27,35]. In some southern locations such as Limbe and Edea, *Ae*. *albopictus* was found to be more prevalent in suburb whereas it is *Ae*. *aegypti* that was found more in downtown. *Ae*. *aegypti* was also the prevalent species in both suburb and downtown sites in Douala located in the coastal region in the southern part of Cameroon. This observation is consistent with a previous report in Douala in 2006 suggesting that the prevailing climate in Douala is not favourable for the propagation of the invading species [37]. All these observations suggest that the differences in the proportions of both species found in different locations in southern Cameroon may probably reflect differences in environmental factors such as climate, vegetation and building density as suggested previously [37].

Due to the fact that both species exploit the same ecological niches and resources (larval habitat, blood source), *Ae*. *aegypti* and *Ae*. *albopictus* have competitive interactions [30,32,55] that may result in the replacement of one species by another in a given environment. Indeed, several studies carried out around the world have shown the changes of the range and abundance of the native species after the introduction of *Ae*. *albopictus* [31,33,34]. In Cameroon, where both species exploit the same types of resources, it is possible that such competitive phenomena are in progress. The mechanisms for the competition are not well known, but many authors believe that it could occur at the pre-imaginal phase and that several factors such as temperature, precipitation, response to symbionts, parasites, predators, and chemical interferences delaying growth could be the main driving forces [30,32]. In addition, other studies demonstrated that mating interference in favour to *Ae*. *albopictus*, called satyrization, is one of the probable cause of the competitive displacement of resident *Ae*. *aegypti* by the invasive *Ae*. *albopictus* where they co-exist [56,57]. On the other hand, the coexistence of *Ae*. *aegypti* and *Ae*. *albopictus* was reported in certain locations in Florida (USA) two decades after competitive displacement [58].

Used tires were the containers mostly found and mostly positive as breeding sites across the country. This is consistent with previous studies in central Africa demonstrating that used tires are the main productive for both *Ae*. *aegypti* and *Ae*. *albopictus* [11,35–37]. The propensity of *Ae*. *aegypti* and *Ae*. *albopictus* to colonize used tires may be due to the fact that these species are native to the forest and breed mainly in natural tree holes, which share the characteristics of tires, as the dark colour and the dark interior provide attractive resting or oviposition site for *Aedes* spp. as previously suggested [35]. Nevertheless, in this study, sampling was targeted mainly at garages and used tire shops to increase the chances of finding the immature stages of *Aedes* spp. It is important to highlight the remarkable presence of tires as they are used to protect water wells in certain locations such as Garoua Boulai, Bertoua, Bankim and Tibati. This important observation could help raise the awareness in the populations of inadvertently providing larval habitats for arbovirus vectors due to this habit potentially helping to fight against the arboviral diseases.

The presence and higher prevalence of *Ae*. *albopictus* in southern part of Cameroon can have a significant impact on the epidemiology of mosquito-borne arboviral diseases since *Ae*. *albopictus* has been found competent to transmit about 22 arboviruses [59]. Interestingly, the emergence of dengue and chikungunya viruses in domesticated environments in central Africa coincides with the introduction of *Ae*. *albopictus* in this area [11,12,24]. In addition, *Ae*. *albopictus* was found infected by zika virus in natural conditions in Gabon in Central Africa [60]. It was also demonstrated that *Ae*. *albopictus* from Bangui in the Central African Republic is able to transmit enzootic chikungunya virus strain [61]. This suggests *Ae*. *albopictus* can serve as bridge to transfer viruses from sylvan area to urban in central Africa whether this species become dominant in wild settings. Further studies assessing the spread of the invading species *Ae*. *albopictus* in sylvan and rural environments are also needed.

The discrepancy observed in the distribution of *Ae*. *albopictus* and *Ae*. *aegypti* across the country notably the restriction of *Ae*. *albopictus* in the southern part suggest that the implementing of vector control programme should take into account the specificity of each area. However, data collected across the country show that a good system of waste management in the domesticated environment including the destroying of the used tires could contribute to reduce the density of both *Ae*. *aegypti* and *Ae*. *albopictus* in Cameroon and indirectly reduce the risk of transmission of diseases transmitted by these mosquitoes.

MtDNA analysis using the COI gene in *Ae*. *albopictus* populations from Cameroon revealed a low polymorphism with only five haplotypes detected across the country. Among these haplotypes, three (H1, H2 and H3) of them have been detected previously in the Republic of the Congo with the same primers [50]. This low polymorphism reported in Cameroon and Republic of the Congo is in accordance with the previous studies using another portion of the COI gene in areas newly colonised by *Ae*. *albopictus* including Central African countries [9,35,62]. It was previously suggested that this low polymorphism is mainly due to the recent introduction of *Ae*. *albopictus* from a founder population [35]. Indeed, *Ae*. *albopictus* was reported for the first time in Cameroon in 1999. Phylogenetic analysis showed that the haplotype sequences from Cameroon are very close to other sequences isolated to the populations originating from China and Congo, suggesting Cameroonian’s and Congolese populations could have the same origin. Meanwhile, the fact that more haplotypes are found in Cameroon suggests that this country could have been the entrance point of *Ae*. *albopictus* in Central Africa potentially through the Port of Douala which is the major one in Central Africa. Primers used in this current study were not the same as those used in the previous study in Cameroon, Central African Republic, and Sao Tome Island. Thus, it was not possible to compare the haplotypes detected in this study with the previous ones detected in Central Africa. Nevertheless, the current results support previous findings suggesting that it is likely that the invading population which colonized Central Africa originated mainly from other tropical regions of the world [9,35,62]. Further studies, including samples from all central African region using other markers such as double digest RAD sequencing, are required to assess the genetic structure and the level of the gene flow between central African *Ae*. *albopictus* populations.

This study shows that for the past 10 years the distribution of *Ae*. *albopictus* is still restricted to southern Cameroon below 6°5’N latitude while *Ae*. *aegypti* is present across the country. This suggests that the prevailing climate in the northern part of Cameroon is not conducive to the invading species *Ae*. *albopictus* in this part of the country. However, the invading species is more prevalent in almost all locations in sympatric with the native species suggesting replacement of native species *Ae*. *aegypti* is ongoing in some locations.

## Supporting information

S1 Table Prevalence of each haplotype detected across Cameroon. (DOCX)
