# Supplementary material for: Update on the geographical distribution and prevalence of Aedes aegypti and Aedes albopictus (Diptera: Culicidae), two major arbovirus vectors in Cameroon
Source: PLoS Negl Trop Dis. 2019 Mar 18;13(3):e0007137. doi: 10.1371/journal.pntd.0007137 (PMC6438584; doi:10.1371/journal.pntd.0007137)
Supplement: S1 Table — (DOCX) [file pntd.0007137.s001.docx]

**S1 Table. Prevalence of each haplotype detected across Cameroon.**

|  |  |  | Haplotype |  |  |  |
| --- | --- | --- | --- | --- | --- | --- |
| Location | H1  n (%) | H2  n (%) | H3  n (%) | H4  n (%) | H5  n (%) | All  n (%) |
| Abong-Mbang | 8 (88.9) | 1 (11.1) | 0 (0.0) | 0 (0.0) | 0 (0.0) | 9 (100.0) |
| Bafang | 12 (92.3) | 1 (7.7) | 0 (0.0) | 0 (0.0) | 0 (0.0) | 13 (100.0) |
| Bafoussam | 14 (100.0) | 0 (0.0) | 0 (0.0) | 0 (0.0) | 0 (0.0) | 14 (100.0) |
| Bamenda | 13 (100.0) | 0 (0.0) | 0 (0.0) | 0 (0.0) | 0 (0.0) | 13 (100.0) |
| Bankim | 14 (100.0) | 0 (0.0) | 0 (0.0) | 0 (0.0) | 0 (0.0) | 14 (100.0) |
| Bertoua | 15 (100.0) | 0 (0.0) | 0 (0.0) | 0 (0.0) | 0 (0.0) | 15 (100.0) |
| Buea | 11 (78.6) | 0 (0.0) | 3 (21.4) | 0 (0.0) | 0 (0.0) | 14 (100.0) |
| Douala | 8 (80.0) | 0 (0.0) | 2 (20.0) | 0 (0.0) | 0 (0.0) | 10 (100.0) |
| Ebolowa | 10 (71.4) | 0 (0.0) | 3 (21.4) | 1 (7.2) | 0 (0.0) | 14 (100.0) |
| Garoua-Boulai | 14 (93.3) | 0 (0.0) | 1 (6.7) | 0 (0.0) | 0 (0.0) | 15 (100.0) |
| Kumba | 6 (46.2) | 6 (46.2) | 1 (7.7) | 0 (0.0) | 0 (0.0) | 13 (100.0) |
| Limbe | 12 (85.7) | 2 (14.3) | 0 (0.0) | 0 (0.0) | 0 (0.0) | 14 (100.0) |
| Melong | 12 (80.0) | 0 (0.0) | 1 (6.7) | 0 (0.0) | 2 (13.3) | 15 (100.0) |
| Tibati | 14 (100.0) | 0 (0.0) | 0 (0.0) | 0 (0.0) | 0 (0.0) | 14 (100.0) |
| Bafia | 5 (35.7) | 8 (57.1) | 1 (7.2) | 0 (0.0) | 0 (0.0) | 14 (100.0) |
| Kribi | 5 (45.5) | 0 (0.0) | 6 (54.5) | 0 (0.0) | 0 (0.0) | 11 (100.0) |
| Yaounde | 7 (50.0) | 2 (14.3) | 5 (35.7) | 0 (0.0) | 0 (0.0) | 14 (100.0) |
| **All** | **180 (79.7)** | **20 (8.8)** | **23 (10.2)** | **1 (0.4)** | **2 (0.9)** | **226 (100.0)** |

**n, number of haplotypes; %, percentage.**
